# Supplementary figures and images for: Dual-stream transformer approach for pain assessment using visual-physiological data modeling (part 2 of 2)
Source: PeerJ Comput Sci. 2025 Sep 3;11:e3158. doi: 10.7717/peerj-cs.3158 (PMC12453799; doi:10.7717/peerj-cs.3158)

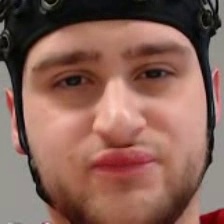

Supplement: Supplemental Information 1 [file peerj-cs-11-3158-s001.zip › ai4pain_samples/cropped/3/H2/keyframe_107.jpg]

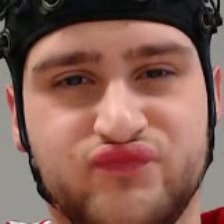

Supplement: Supplemental Information 1 [file peerj-cs-11-3158-s001.zip › ai4pain_samples/cropped/3/H2/keyframe_114.jpg]

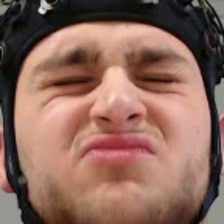

Supplement: Supplemental Information 1 [file peerj-cs-11-3158-s001.zip › ai4pain_samples/cropped/3/H2/keyframe_12.jpg]

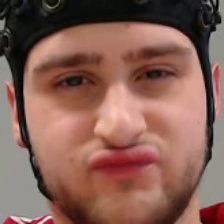

Supplement: Supplemental Information 1 [file peerj-cs-11-3158-s001.zip › ai4pain_samples/cropped/3/H2/keyframe_124.jpg]

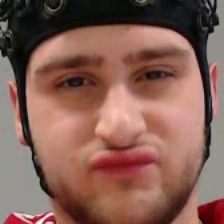

Supplement: Supplemental Information 1 [file peerj-cs-11-3158-s001.zip › ai4pain_samples/cropped/3/H2/keyframe_125.jpg]

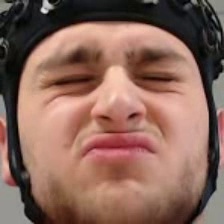

Supplement: Supplemental Information 1 [file peerj-cs-11-3158-s001.zip › ai4pain_samples/cropped/3/H2/keyframe_15.jpg]

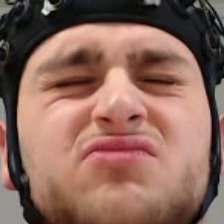

Supplement: Supplemental Information 1 [file peerj-cs-11-3158-s001.zip › ai4pain_samples/cropped/3/H2/keyframe_18.jpg]

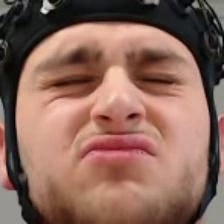

Supplement: Supplemental Information 1 [file peerj-cs-11-3158-s001.zip › ai4pain_samples/cropped/3/H2/keyframe_19.jpg]

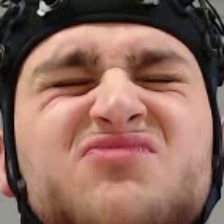

Supplement: Supplemental Information 1 [file peerj-cs-11-3158-s001.zip › ai4pain_samples/cropped/3/H2/keyframe_2.jpg]

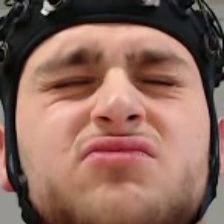

Supplement: Supplemental Information 1 [file peerj-cs-11-3158-s001.zip › ai4pain_samples/cropped/3/H2/keyframe_20.jpg]

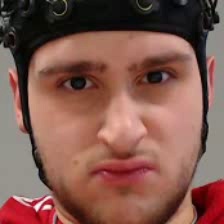

Supplement: Supplemental Information 1 [file peerj-cs-11-3158-s001.zip › ai4pain_samples/cropped/3/H2/keyframe_204.jpg]

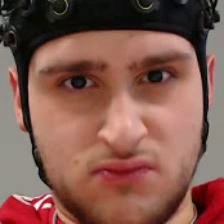

Supplement: Supplemental Information 1 [file peerj-cs-11-3158-s001.zip › ai4pain_samples/cropped/3/H2/keyframe_206.jpg]

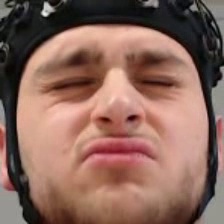

Supplement: Supplemental Information 1 [file peerj-cs-11-3158-s001.zip › ai4pain_samples/cropped/3/H2/keyframe_22.jpg]

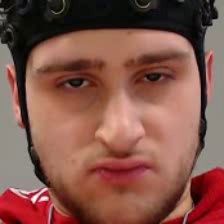

Supplement: Supplemental Information 1 [file peerj-cs-11-3158-s001.zip › ai4pain_samples/cropped/3/H2/keyframe_228.jpg]

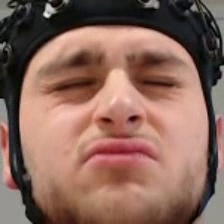

Supplement: Supplemental Information 1 [file peerj-cs-11-3158-s001.zip › ai4pain_samples/cropped/3/H2/keyframe_23.jpg]

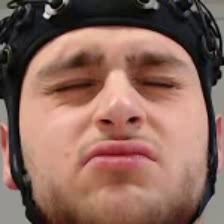

Supplement: Supplemental Information 1 [file peerj-cs-11-3158-s001.zip › ai4pain_samples/cropped/3/H2/keyframe_24.jpg]

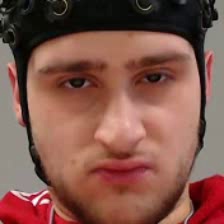

Supplement: Supplemental Information 1 [file peerj-cs-11-3158-s001.zip › ai4pain_samples/cropped/3/H2/keyframe_240.jpg]

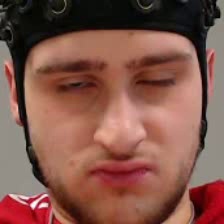

Supplement: Supplemental Information 1 [file peerj-cs-11-3158-s001.zip › ai4pain_samples/cropped/3/H2/keyframe_252.jpg]

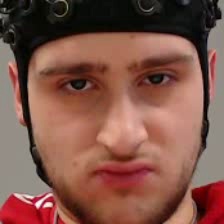

Supplement: Supplemental Information 1 [file peerj-cs-11-3158-s001.zip › ai4pain_samples/cropped/3/H2/keyframe_264.jpg]

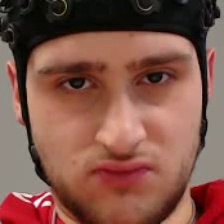

Supplement: Supplemental Information 1 [file peerj-cs-11-3158-s001.zip › ai4pain_samples/cropped/3/H2/keyframe_266.jpg]

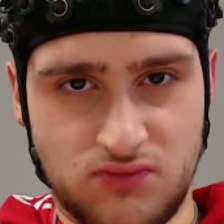

Supplement: Supplemental Information 1 [file peerj-cs-11-3158-s001.zip › ai4pain_samples/cropped/3/H2/keyframe_267.jpg]

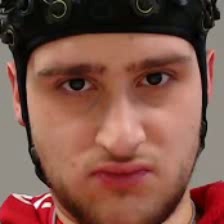

Supplement: Supplemental Information 1 [file peerj-cs-11-3158-s001.zip › ai4pain_samples/cropped/3/H2/keyframe_276.jpg]

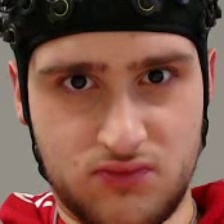

Supplement: Supplemental Information 1 [file peerj-cs-11-3158-s001.zip › ai4pain_samples/cropped/3/H2/keyframe_287.jpg]

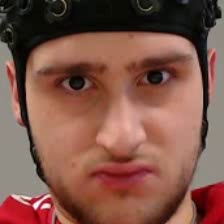

Supplement: Supplemental Information 1 [file peerj-cs-11-3158-s001.zip › ai4pain_samples/cropped/3/H2/keyframe_288.jpg]

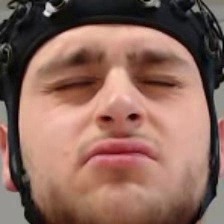

Supplement: Supplemental Information 1 [file peerj-cs-11-3158-s001.zip › ai4pain_samples/cropped/3/H2/keyframe_29.jpg]

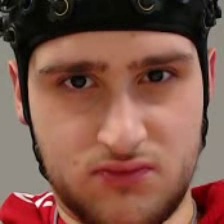

Supplement: Supplemental Information 1 [file peerj-cs-11-3158-s001.zip › ai4pain_samples/cropped/3/H2/keyframe_299.jpg]

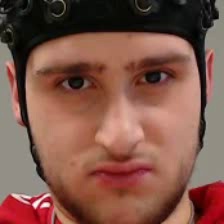

Supplement: Supplemental Information 1 [file peerj-cs-11-3158-s001.zip › ai4pain_samples/cropped/3/H2/keyframe_300.jpg]

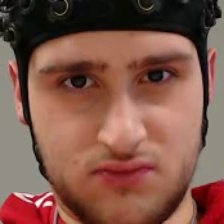

Supplement: Supplemental Information 1 [file peerj-cs-11-3158-s001.zip › ai4pain_samples/cropped/3/H2/keyframe_304.jpg]

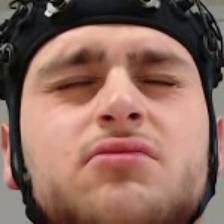

Supplement: Supplemental Information 1 [file peerj-cs-11-3158-s001.zip › ai4pain_samples/cropped/3/H2/keyframe_31.jpg]

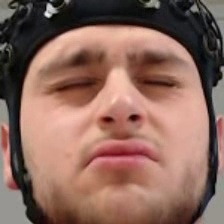

Supplement: Supplemental Information 1 [file peerj-cs-11-3158-s001.zip › ai4pain_samples/cropped/3/H2/keyframe_33.jpg]

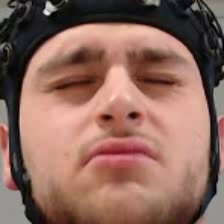

Supplement: Supplemental Information 1 [file peerj-cs-11-3158-s001.zip › ai4pain_samples/cropped/3/H2/keyframe_36.jpg]

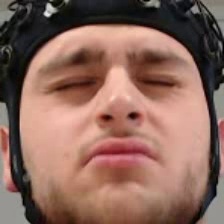

Supplement: Supplemental Information 1 [file peerj-cs-11-3158-s001.zip › ai4pain_samples/cropped/3/H2/keyframe_37.jpg]

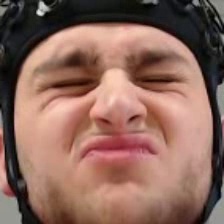

Supplement: Supplemental Information 1 [file peerj-cs-11-3158-s001.zip › ai4pain_samples/cropped/3/H2/keyframe_4.jpg]

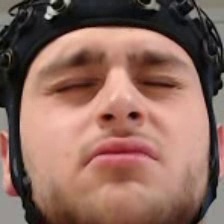

Supplement: Supplemental Information 1 [file peerj-cs-11-3158-s001.zip › ai4pain_samples/cropped/3/H2/keyframe_42.jpg]

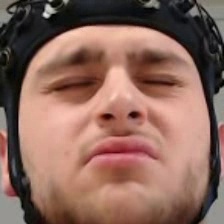

Supplement: Supplemental Information 1 [file peerj-cs-11-3158-s001.zip › ai4pain_samples/cropped/3/H2/keyframe_43.jpg]

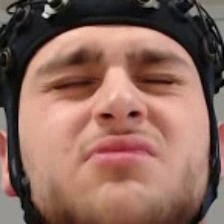

Supplement: Supplemental Information 1 [file peerj-cs-11-3158-s001.zip › ai4pain_samples/cropped/3/H2/keyframe_44.jpg]

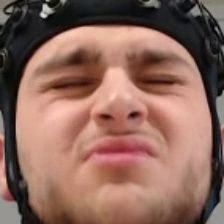

Supplement: Supplemental Information 1 [file peerj-cs-11-3158-s001.zip › ai4pain_samples/cropped/3/H2/keyframe_45.jpg]

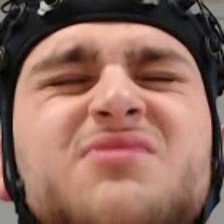

Supplement: Supplemental Information 1 [file peerj-cs-11-3158-s001.zip › ai4pain_samples/cropped/3/H2/keyframe_46.jpg]

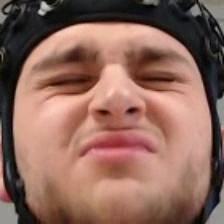

Supplement: Supplemental Information 1 [file peerj-cs-11-3158-s001.zip › ai4pain_samples/cropped/3/H2/keyframe_47.jpg]

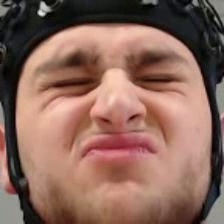

Supplement: Supplemental Information 1 [file peerj-cs-11-3158-s001.zip › ai4pain_samples/cropped/3/H2/keyframe_5.jpg]

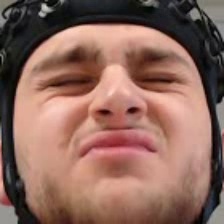

Supplement: Supplemental Information 1 [file peerj-cs-11-3158-s001.zip › ai4pain_samples/cropped/3/H2/keyframe_50.jpg]

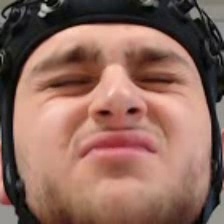

Supplement: Supplemental Information 1 [file peerj-cs-11-3158-s001.zip › ai4pain_samples/cropped/3/H2/keyframe_51.jpg]

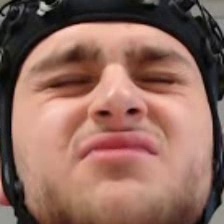

Supplement: Supplemental Information 1 [file peerj-cs-11-3158-s001.zip › ai4pain_samples/cropped/3/H2/keyframe_52.jpg]

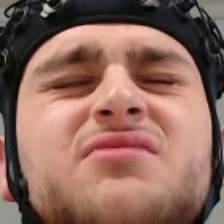

Supplement: Supplemental Information 1 [file peerj-cs-11-3158-s001.zip › ai4pain_samples/cropped/3/H2/keyframe_53.jpg]

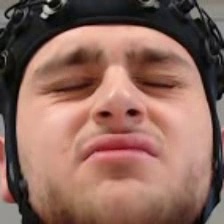

Supplement: Supplemental Information 1 [file peerj-cs-11-3158-s001.zip › ai4pain_samples/cropped/3/H2/keyframe_54.jpg]

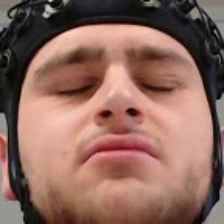

Supplement: Supplemental Information 1 [file peerj-cs-11-3158-s001.zip › ai4pain_samples/cropped/3/H2/keyframe_56.jpg]

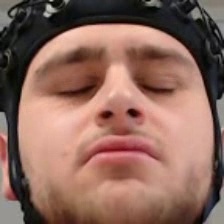

Supplement: Supplemental Information 1 [file peerj-cs-11-3158-s001.zip › ai4pain_samples/cropped/3/H2/keyframe_57.jpg]

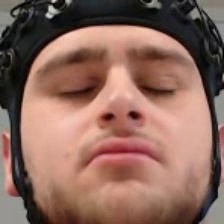

Supplement: Supplemental Information 1 [file peerj-cs-11-3158-s001.zip › ai4pain_samples/cropped/3/H2/keyframe_59.jpg]

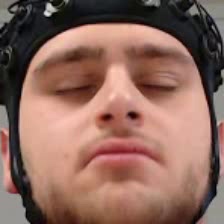

Supplement: Supplemental Information 1 [file peerj-cs-11-3158-s001.zip › ai4pain_samples/cropped/3/H2/keyframe_60.jpg]

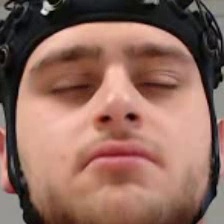

Supplement: Supplemental Information 1 [file peerj-cs-11-3158-s001.zip › ai4pain_samples/cropped/3/H2/keyframe_61.jpg]

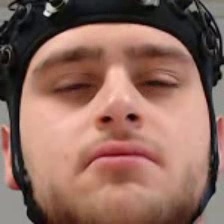

Supplement: Supplemental Information 1 [file peerj-cs-11-3158-s001.zip › ai4pain_samples/cropped/3/H2/keyframe_62.jpg]

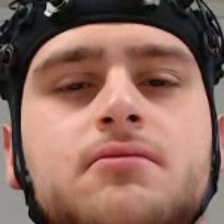

Supplement: Supplemental Information 1 [file peerj-cs-11-3158-s001.zip › ai4pain_samples/cropped/3/H2/keyframe_63.jpg]

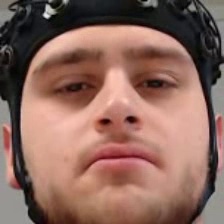

Supplement: Supplemental Information 1 [file peerj-cs-11-3158-s001.zip › ai4pain_samples/cropped/3/H2/keyframe_64.jpg]

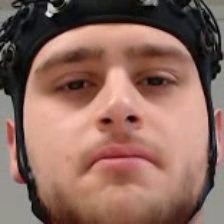

Supplement: Supplemental Information 1 [file peerj-cs-11-3158-s001.zip › ai4pain_samples/cropped/3/H2/keyframe_67.jpg]

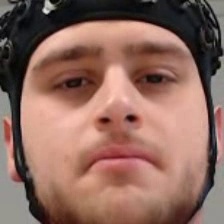

Supplement: Supplemental Information 1 [file peerj-cs-11-3158-s001.zip › ai4pain_samples/cropped/3/H2/keyframe_68.jpg]

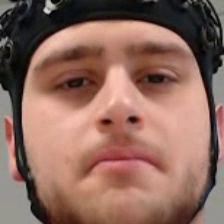

Supplement: Supplemental Information 1 [file peerj-cs-11-3158-s001.zip › ai4pain_samples/cropped/3/H2/keyframe_69.jpg]

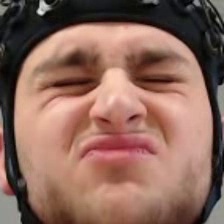

Supplement: Supplemental Information 1 [file peerj-cs-11-3158-s001.zip › ai4pain_samples/cropped/3/H2/keyframe_7.jpg]

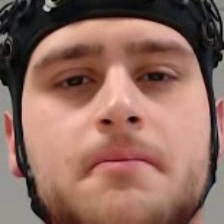

Supplement: Supplemental Information 1 [file peerj-cs-11-3158-s001.zip › ai4pain_samples/cropped/3/H2/keyframe_71.jpg]

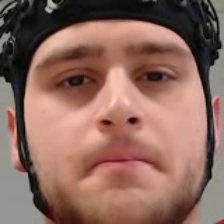

Supplement: Supplemental Information 1 [file peerj-cs-11-3158-s001.zip › ai4pain_samples/cropped/3/H2/keyframe_77.jpg]

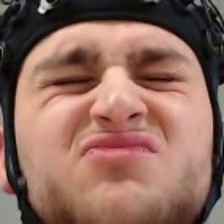

Supplement: Supplemental Information 1 [file peerj-cs-11-3158-s001.zip › ai4pain_samples/cropped/3/H2/keyframe_8.jpg]

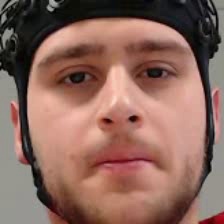

Supplement: Supplemental Information 1 [file peerj-cs-11-3158-s001.zip › ai4pain_samples/cropped/3/H2/keyframe_84.jpg]

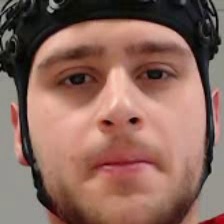

Supplement: Supplemental Information 1 [file peerj-cs-11-3158-s001.zip › ai4pain_samples/cropped/3/H2/keyframe_85.jpg]

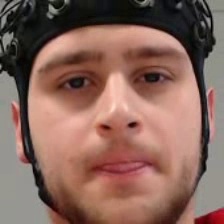

Supplement: Supplemental Information 1 [file peerj-cs-11-3158-s001.zip › ai4pain_samples/cropped/3/H2/keyframe_89.jpg]

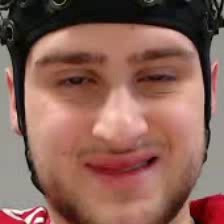

Supplement: Supplemental Information 1 [file peerj-cs-11-3158-s001.zip › ai4pain_samples/cropped/3/H3/keyframe_0.jpg]

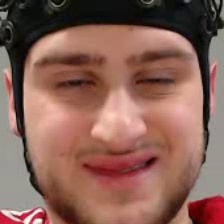

Supplement: Supplemental Information 1 [file peerj-cs-11-3158-s001.zip › ai4pain_samples/cropped/3/H3/keyframe_1.jpg]

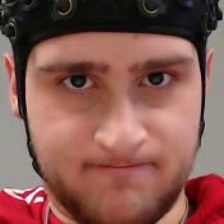

Supplement: Supplemental Information 1 [file peerj-cs-11-3158-s001.zip › ai4pain_samples/cropped/3/H3/keyframe_105.jpg]

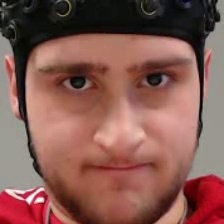

Supplement: Supplemental Information 1 [file peerj-cs-11-3158-s001.zip › ai4pain_samples/cropped/3/H3/keyframe_109.jpg]

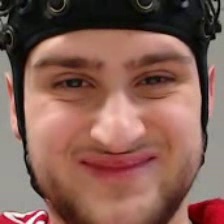

Supplement: Supplemental Information 1 [file peerj-cs-11-3158-s001.zip › ai4pain_samples/cropped/3/H3/keyframe_11.jpg]

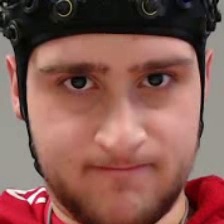

Supplement: Supplemental Information 1 [file peerj-cs-11-3158-s001.zip › ai4pain_samples/cropped/3/H3/keyframe_110.jpg]

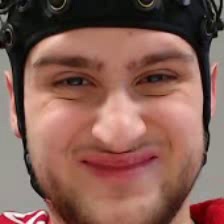

Supplement: Supplemental Information 1 [file peerj-cs-11-3158-s001.zip › ai4pain_samples/cropped/3/H3/keyframe_12.jpg]

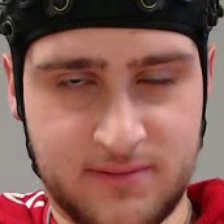

Supplement: Supplemental Information 1 [file peerj-cs-11-3158-s001.zip › ai4pain_samples/cropped/3/H3/keyframe_124.jpg]

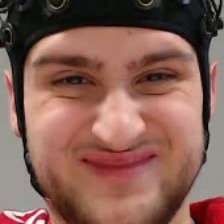

Supplement: Supplemental Information 1 [file peerj-cs-11-3158-s001.zip › ai4pain_samples/cropped/3/H3/keyframe_13.jpg]

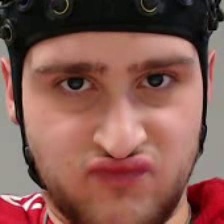

Supplement: Supplemental Information 1 [file peerj-cs-11-3158-s001.zip › ai4pain_samples/cropped/3/H3/keyframe_155.jpg]

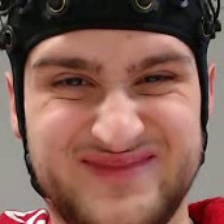

Supplement: Supplemental Information 1 [file peerj-cs-11-3158-s001.zip › ai4pain_samples/cropped/3/H3/keyframe_16.jpg]

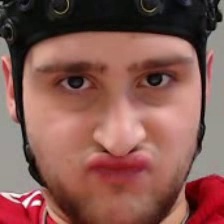

Supplement: Supplemental Information 1 [file peerj-cs-11-3158-s001.zip › ai4pain_samples/cropped/3/H3/keyframe_164.jpg]

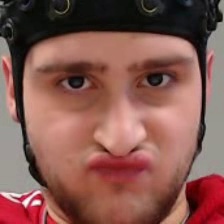

Supplement: Supplemental Information 1 [file peerj-cs-11-3158-s001.zip › ai4pain_samples/cropped/3/H3/keyframe_167.jpg]

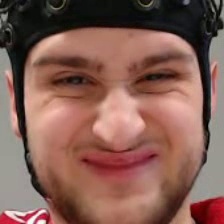

Supplement: Supplemental Information 1 [file peerj-cs-11-3158-s001.zip › ai4pain_samples/cropped/3/H3/keyframe_17.jpg]

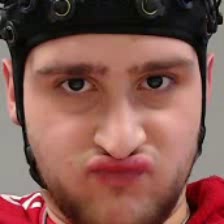

Supplement: Supplemental Information 1 [file peerj-cs-11-3158-s001.zip › ai4pain_samples/cropped/3/H3/keyframe_180.jpg]

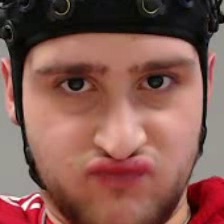

Supplement: Supplemental Information 1 [file peerj-cs-11-3158-s001.zip › ai4pain_samples/cropped/3/H3/keyframe_182.jpg]

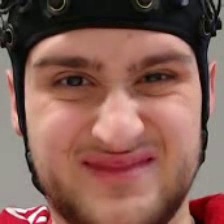

Supplement: Supplemental Information 1 [file peerj-cs-11-3158-s001.zip › ai4pain_samples/cropped/3/H3/keyframe_22.jpg]

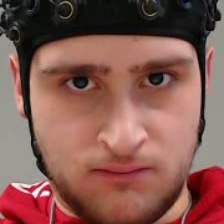

Supplement: Supplemental Information 1 [file peerj-cs-11-3158-s001.zip › ai4pain_samples/cropped/3/H3/keyframe_273.jpg]

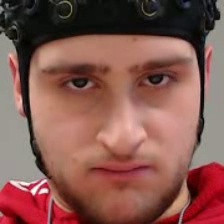

Supplement: Supplemental Information 1 [file peerj-cs-11-3158-s001.zip › ai4pain_samples/cropped/3/H3/keyframe_293.jpg]

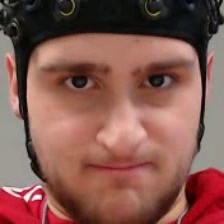

Supplement: Supplemental Information 1 [file peerj-cs-11-3158-s001.zip › ai4pain_samples/cropped/3/H3/keyframe_41.jpg]

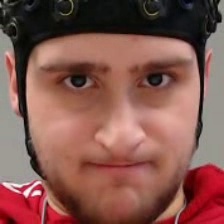

Supplement: Supplemental Information 1 [file peerj-cs-11-3158-s001.zip › ai4pain_samples/cropped/3/H3/keyframe_47.jpg]

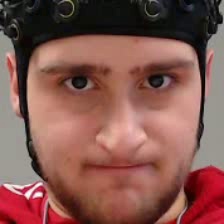

Supplement: Supplemental Information 1 [file peerj-cs-11-3158-s001.zip › ai4pain_samples/cropped/3/H3/keyframe_48.jpg]

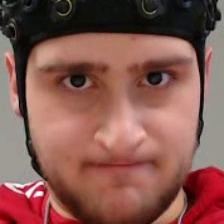

Supplement: Supplemental Information 1 [file peerj-cs-11-3158-s001.zip › ai4pain_samples/cropped/3/H3/keyframe_55.jpg]

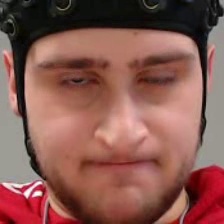

Supplement: Supplemental Information 1 [file peerj-cs-11-3158-s001.zip › ai4pain_samples/cropped/3/H3/keyframe_65.jpg]

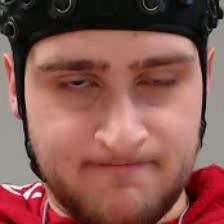

Supplement: Supplemental Information 1 [file peerj-cs-11-3158-s001.zip › ai4pain_samples/cropped/3/H3/keyframe_72.jpg]

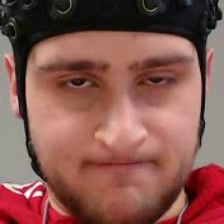

Supplement: Supplemental Information 1 [file peerj-cs-11-3158-s001.zip › ai4pain_samples/cropped/3/H3/keyframe_73.jpg]

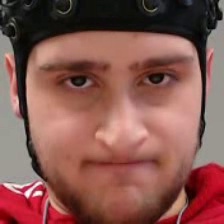

Supplement: Supplemental Information 1 [file peerj-cs-11-3158-s001.zip › ai4pain_samples/cropped/3/H3/keyframe_76.jpg]

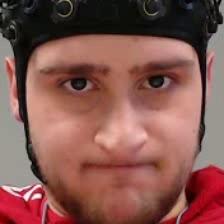

Supplement: Supplemental Information 1 [file peerj-cs-11-3158-s001.zip › ai4pain_samples/cropped/3/H3/keyframe_84.jpg]

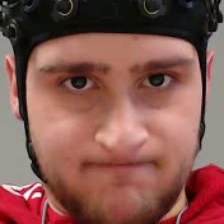

Supplement: Supplemental Information 1 [file peerj-cs-11-3158-s001.zip › ai4pain_samples/cropped/3/H3/keyframe_85.jpg]

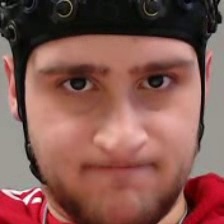

Supplement: Supplemental Information 1 [file peerj-cs-11-3158-s001.zip › ai4pain_samples/cropped/3/H3/keyframe_92.jpg]

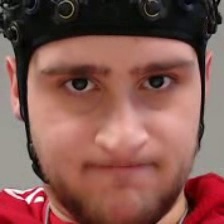

Supplement: Supplemental Information 1 [file peerj-cs-11-3158-s001.zip › ai4pain_samples/cropped/3/H3/keyframe_94.jpg]

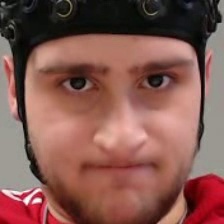

Supplement: Supplemental Information 1 [file peerj-cs-11-3158-s001.zip › ai4pain_samples/cropped/3/H3/keyframe_95.jpg]

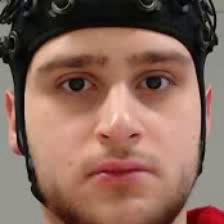

Supplement: Supplemental Information 1 [file peerj-cs-11-3158-s001.zip › ai4pain_samples/cropped/3/L1/keyframe_0.jpg]

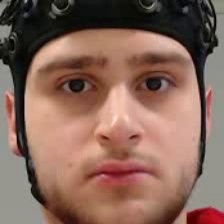

Supplement: Supplemental Information 1 [file peerj-cs-11-3158-s001.zip › ai4pain_samples/cropped/3/L1/keyframe_1.jpg]

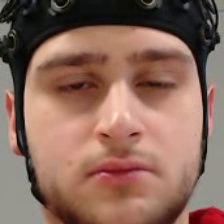

Supplement: Supplemental Information 1 [file peerj-cs-11-3158-s001.zip › ai4pain_samples/cropped/3/L1/keyframe_10.jpg]

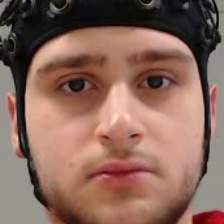

Supplement: Supplemental Information 1 [file peerj-cs-11-3158-s001.zip › ai4pain_samples/cropped/3/L1/keyframe_100.jpg]

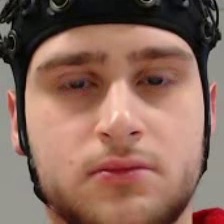

Supplement: Supplemental Information 1 [file peerj-cs-11-3158-s001.zip › ai4pain_samples/cropped/3/L1/keyframe_103.jpg]
