# Supplementary figures and images for: Dual-stream transformer approach for pain assessment using visual-physiological data modeling (part 1 of 2)
Source: PeerJ Comput Sci. 2025 Sep 3;11:e3158. doi: 10.7717/peerj-cs.3158 (PMC12453799; doi:10.7717/peerj-cs.3158)

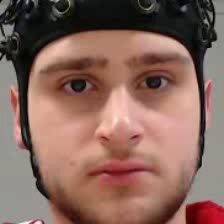

Supplement: Supplemental Information 1 [file peerj-cs-11-3158-s001.zip › ai4pain_samples/cropped/3/B1/keyframe_0.jpg]

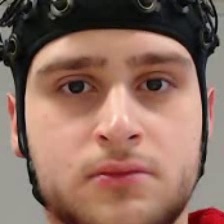

Supplement: Supplemental Information 1 [file peerj-cs-11-3158-s001.zip › ai4pain_samples/cropped/3/B1/keyframe_1000.jpg]

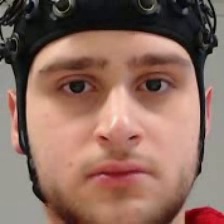

Supplement: Supplemental Information 1 [file peerj-cs-11-3158-s001.zip › ai4pain_samples/cropped/3/B1/keyframe_1048.jpg]

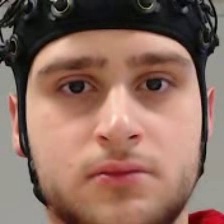

Supplement: Supplemental Information 1 [file peerj-cs-11-3158-s001.zip › ai4pain_samples/cropped/3/B1/keyframe_1063.jpg]

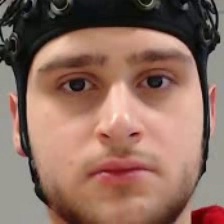

Supplement: Supplemental Information 1 [file peerj-cs-11-3158-s001.zip › ai4pain_samples/cropped/3/B1/keyframe_1135.jpg]

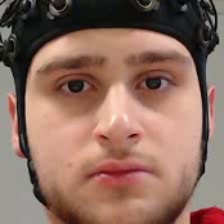

Supplement: Supplemental Information 1 [file peerj-cs-11-3158-s001.zip › ai4pain_samples/cropped/3/B1/keyframe_1140.jpg]

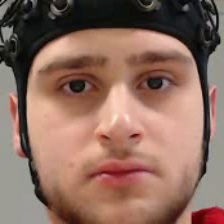

Supplement: Supplemental Information 1 [file peerj-cs-11-3158-s001.zip › ai4pain_samples/cropped/3/B1/keyframe_1141.jpg]

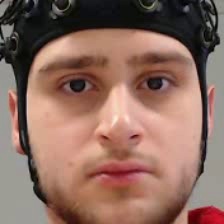

Supplement: Supplemental Information 1 [file peerj-cs-11-3158-s001.zip › ai4pain_samples/cropped/3/B1/keyframe_1224.jpg]

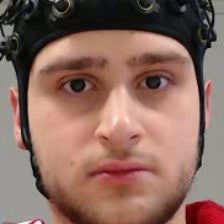

Supplement: Supplemental Information 1 [file peerj-cs-11-3158-s001.zip › ai4pain_samples/cropped/3/B1/keyframe_132.jpg]

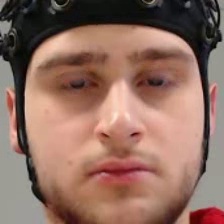

Supplement: Supplemental Information 1 [file peerj-cs-11-3158-s001.zip › ai4pain_samples/cropped/3/B1/keyframe_1346.jpg]

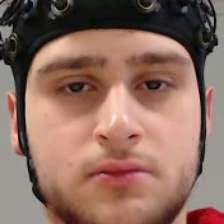

Supplement: Supplemental Information 1 [file peerj-cs-11-3158-s001.zip › ai4pain_samples/cropped/3/B1/keyframe_1351.jpg]

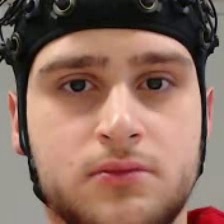

Supplement: Supplemental Information 1 [file peerj-cs-11-3158-s001.zip › ai4pain_samples/cropped/3/B1/keyframe_1371.jpg]

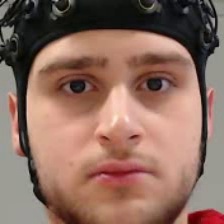

Supplement: Supplemental Information 1 [file peerj-cs-11-3158-s001.zip › ai4pain_samples/cropped/3/B1/keyframe_1455.jpg]

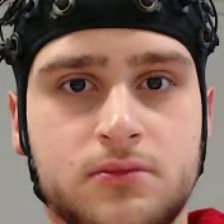

Supplement: Supplemental Information 1 [file peerj-cs-11-3158-s001.zip › ai4pain_samples/cropped/3/B1/keyframe_1461.jpg]

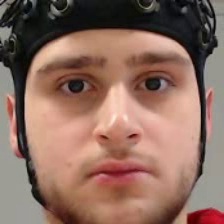

Supplement: Supplemental Information 1 [file peerj-cs-11-3158-s001.zip › ai4pain_samples/cropped/3/B1/keyframe_1493.jpg]

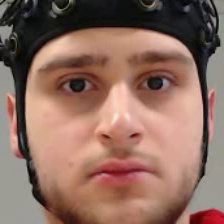

Supplement: Supplemental Information 1 [file peerj-cs-11-3158-s001.zip › ai4pain_samples/cropped/3/B1/keyframe_1506.jpg]

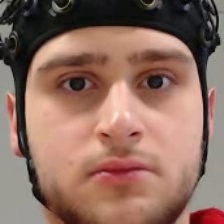

Supplement: Supplemental Information 1 [file peerj-cs-11-3158-s001.zip › ai4pain_samples/cropped/3/B1/keyframe_1509.jpg]

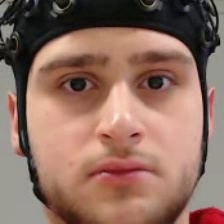

Supplement: Supplemental Information 1 [file peerj-cs-11-3158-s001.zip › ai4pain_samples/cropped/3/B1/keyframe_1521.jpg]

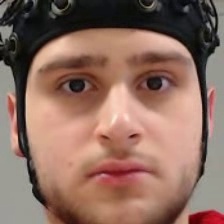

Supplement: Supplemental Information 1 [file peerj-cs-11-3158-s001.zip › ai4pain_samples/cropped/3/B1/keyframe_1547.jpg]

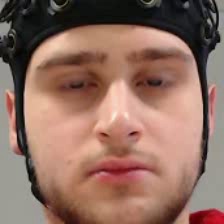

Supplement: Supplemental Information 1 [file peerj-cs-11-3158-s001.zip › ai4pain_samples/cropped/3/B1/keyframe_1560.jpg]

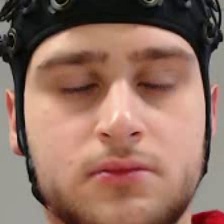

Supplement: Supplemental Information 1 [file peerj-cs-11-3158-s001.zip › ai4pain_samples/cropped/3/B1/keyframe_1561.jpg]

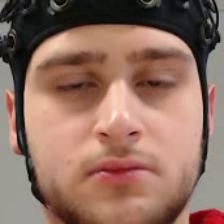

Supplement: Supplemental Information 1 [file peerj-cs-11-3158-s001.zip › ai4pain_samples/cropped/3/B1/keyframe_1563.jpg]

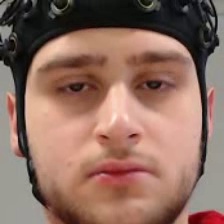

Supplement: Supplemental Information 1 [file peerj-cs-11-3158-s001.zip › ai4pain_samples/cropped/3/B1/keyframe_1564.jpg]

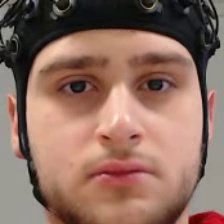

Supplement: Supplemental Information 1 [file peerj-cs-11-3158-s001.zip › ai4pain_samples/cropped/3/B1/keyframe_1582.jpg]

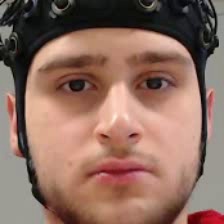

Supplement: Supplemental Information 1 [file peerj-cs-11-3158-s001.zip › ai4pain_samples/cropped/3/B1/keyframe_1584.jpg]

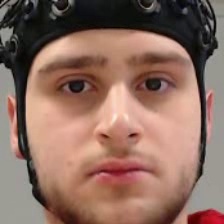

Supplement: Supplemental Information 1 [file peerj-cs-11-3158-s001.zip › ai4pain_samples/cropped/3/B1/keyframe_1612.jpg]

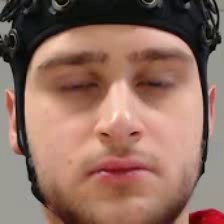

Supplement: Supplemental Information 1 [file peerj-cs-11-3158-s001.zip › ai4pain_samples/cropped/3/B1/keyframe_1656.jpg]

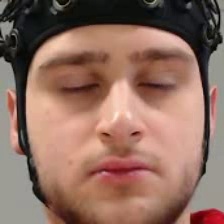

Supplement: Supplemental Information 1 [file peerj-cs-11-3158-s001.zip › ai4pain_samples/cropped/3/B1/keyframe_1657.jpg]

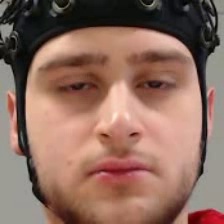

Supplement: Supplemental Information 1 [file peerj-cs-11-3158-s001.zip › ai4pain_samples/cropped/3/B1/keyframe_1659.jpg]

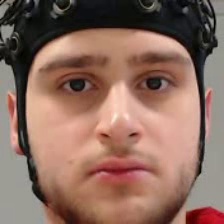

Supplement: Supplemental Information 1 [file peerj-cs-11-3158-s001.zip › ai4pain_samples/cropped/3/B1/keyframe_1695.jpg]

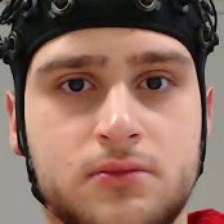

Supplement: Supplemental Information 1 [file peerj-cs-11-3158-s001.zip › ai4pain_samples/cropped/3/B1/keyframe_1757.jpg]

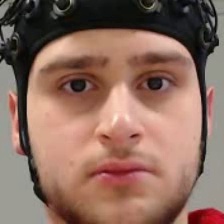

Supplement: Supplemental Information 1 [file peerj-cs-11-3158-s001.zip › ai4pain_samples/cropped/3/B1/keyframe_1765.jpg]

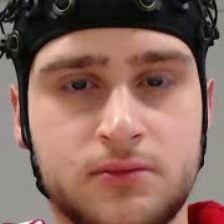

Supplement: Supplemental Information 1 [file peerj-cs-11-3158-s001.zip › ai4pain_samples/cropped/3/B1/keyframe_2.jpg]

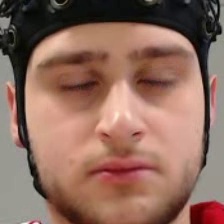

Supplement: Supplemental Information 1 [file peerj-cs-11-3158-s001.zip › ai4pain_samples/cropped/3/B1/keyframe_281.jpg]

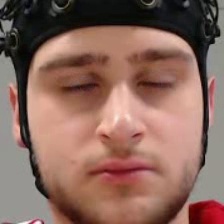

Supplement: Supplemental Information 1 [file peerj-cs-11-3158-s001.zip › ai4pain_samples/cropped/3/B1/keyframe_3.jpg]

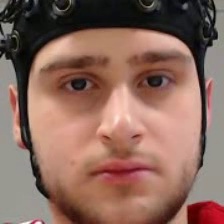

Supplement: Supplemental Information 1 [file peerj-cs-11-3158-s001.zip › ai4pain_samples/cropped/3/B1/keyframe_306.jpg]

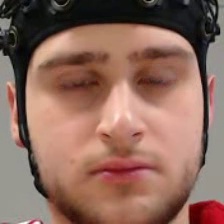

Supplement: Supplemental Information 1 [file peerj-cs-11-3158-s001.zip › ai4pain_samples/cropped/3/B1/keyframe_307.jpg]

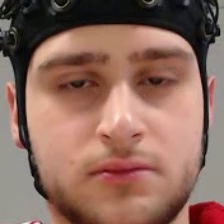

Supplement: Supplemental Information 1 [file peerj-cs-11-3158-s001.zip › ai4pain_samples/cropped/3/B1/keyframe_310.jpg]

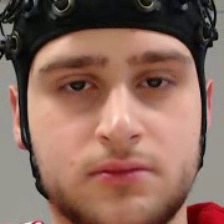

Supplement: Supplemental Information 1 [file peerj-cs-11-3158-s001.zip › ai4pain_samples/cropped/3/B1/keyframe_311.jpg]

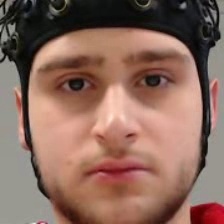

Supplement: Supplemental Information 1 [file peerj-cs-11-3158-s001.zip › ai4pain_samples/cropped/3/B1/keyframe_316.jpg]

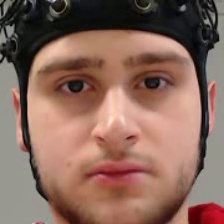

Supplement: Supplemental Information 1 [file peerj-cs-11-3158-s001.zip › ai4pain_samples/cropped/3/B1/keyframe_340.jpg]

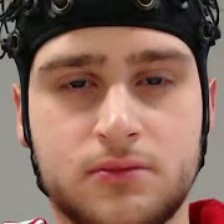

Supplement: Supplemental Information 1 [file peerj-cs-11-3158-s001.zip › ai4pain_samples/cropped/3/B1/keyframe_357.jpg]

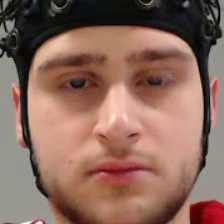

Supplement: Supplemental Information 1 [file peerj-cs-11-3158-s001.zip › ai4pain_samples/cropped/3/B1/keyframe_363.jpg]

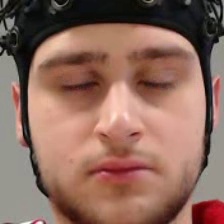

Supplement: Supplemental Information 1 [file peerj-cs-11-3158-s001.zip › ai4pain_samples/cropped/3/B1/keyframe_364.jpg]

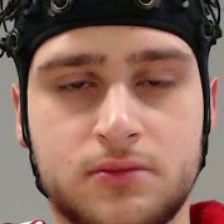

Supplement: Supplemental Information 1 [file peerj-cs-11-3158-s001.zip › ai4pain_samples/cropped/3/B1/keyframe_366.jpg]

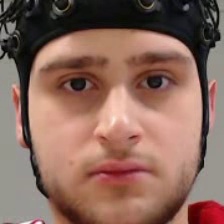

Supplement: Supplemental Information 1 [file peerj-cs-11-3158-s001.zip › ai4pain_samples/cropped/3/B1/keyframe_464.jpg]

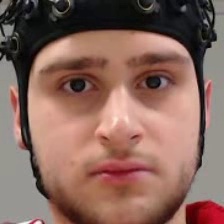

Supplement: Supplemental Information 1 [file peerj-cs-11-3158-s001.zip › ai4pain_samples/cropped/3/B1/keyframe_54.jpg]

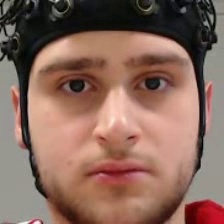

Supplement: Supplemental Information 1 [file peerj-cs-11-3158-s001.zip › ai4pain_samples/cropped/3/B1/keyframe_547.jpg]

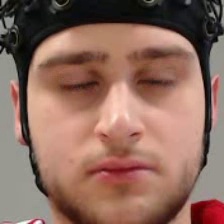

Supplement: Supplemental Information 1 [file peerj-cs-11-3158-s001.zip › ai4pain_samples/cropped/3/B1/keyframe_590.jpg]

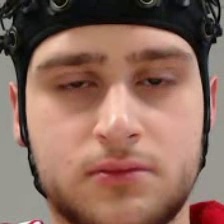

Supplement: Supplemental Information 1 [file peerj-cs-11-3158-s001.zip › ai4pain_samples/cropped/3/B1/keyframe_593.jpg]

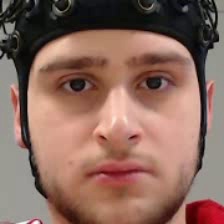

Supplement: Supplemental Information 1 [file peerj-cs-11-3158-s001.zip › ai4pain_samples/cropped/3/B1/keyframe_636.jpg]

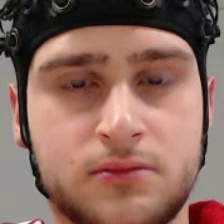

Supplement: Supplemental Information 1 [file peerj-cs-11-3158-s001.zip › ai4pain_samples/cropped/3/B1/keyframe_65.jpg]

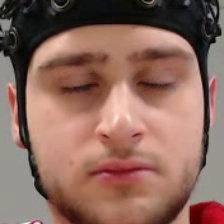

Supplement: Supplemental Information 1 [file peerj-cs-11-3158-s001.zip › ai4pain_samples/cropped/3/B1/keyframe_66.jpg]

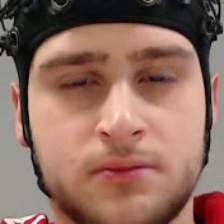

Supplement: Supplemental Information 1 [file peerj-cs-11-3158-s001.zip › ai4pain_samples/cropped/3/B1/keyframe_689.jpg]

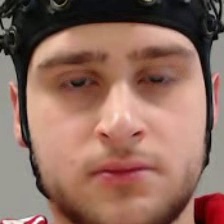

Supplement: Supplemental Information 1 [file peerj-cs-11-3158-s001.zip › ai4pain_samples/cropped/3/B1/keyframe_695.jpg]

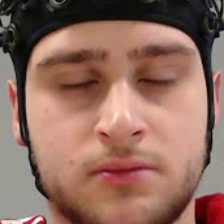

Supplement: Supplemental Information 1 [file peerj-cs-11-3158-s001.zip › ai4pain_samples/cropped/3/B1/keyframe_696.jpg]

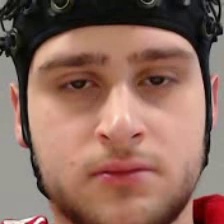

Supplement: Supplemental Information 1 [file peerj-cs-11-3158-s001.zip › ai4pain_samples/cropped/3/B1/keyframe_699.jpg]

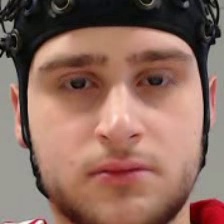

Supplement: Supplemental Information 1 [file peerj-cs-11-3158-s001.zip › ai4pain_samples/cropped/3/B1/keyframe_731.jpg]

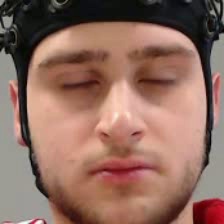

Supplement: Supplemental Information 1 [file peerj-cs-11-3158-s001.zip › ai4pain_samples/cropped/3/B1/keyframe_732.jpg]

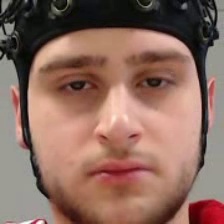

Supplement: Supplemental Information 1 [file peerj-cs-11-3158-s001.zip › ai4pain_samples/cropped/3/B1/keyframe_736.jpg]

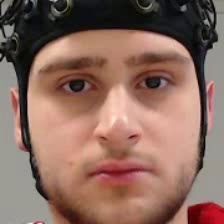

Supplement: Supplemental Information 1 [file peerj-cs-11-3158-s001.zip › ai4pain_samples/cropped/3/B1/keyframe_768.jpg]

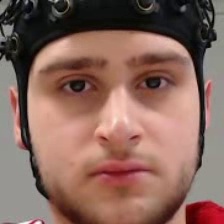

Supplement: Supplemental Information 1 [file peerj-cs-11-3158-s001.zip › ai4pain_samples/cropped/3/B1/keyframe_8.jpg]

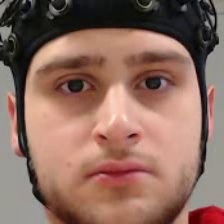

Supplement: Supplemental Information 1 [file peerj-cs-11-3158-s001.zip › ai4pain_samples/cropped/3/B1/keyframe_856.jpg]

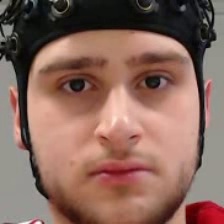

Supplement: Supplemental Information 1 [file peerj-cs-11-3158-s001.zip › ai4pain_samples/cropped/3/B1/keyframe_89.jpg]

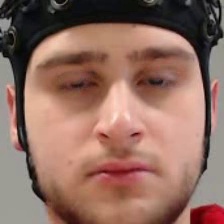

Supplement: Supplemental Information 1 [file peerj-cs-11-3158-s001.zip › ai4pain_samples/cropped/3/B1/keyframe_890.jpg]

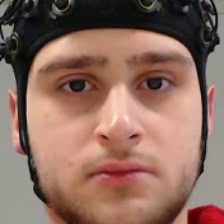

Supplement: Supplemental Information 1 [file peerj-cs-11-3158-s001.zip › ai4pain_samples/cropped/3/B1/keyframe_984.jpg]

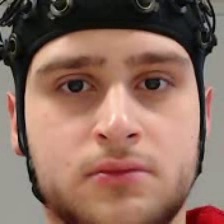

Supplement: Supplemental Information 1 [file peerj-cs-11-3158-s001.zip › ai4pain_samples/cropped/3/B1/keyframe_999.jpg]

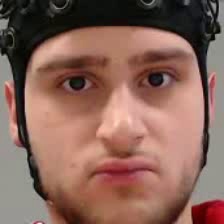

Supplement: Supplemental Information 1 [file peerj-cs-11-3158-s001.zip › ai4pain_samples/cropped/3/H1/keyframe_0.jpg]

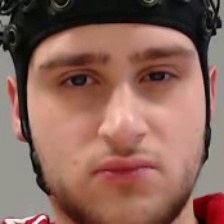

Supplement: Supplemental Information 1 [file peerj-cs-11-3158-s001.zip › ai4pain_samples/cropped/3/H1/keyframe_102.jpg]

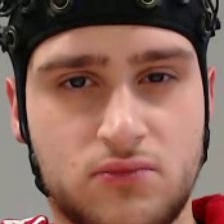

Supplement: Supplemental Information 1 [file peerj-cs-11-3158-s001.zip › ai4pain_samples/cropped/3/H1/keyframe_104.jpg]

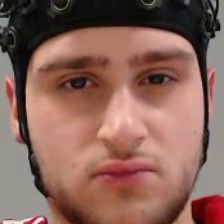

Supplement: Supplemental Information 1 [file peerj-cs-11-3158-s001.zip › ai4pain_samples/cropped/3/H1/keyframe_114.jpg]

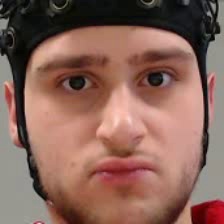

Supplement: Supplemental Information 1 [file peerj-cs-11-3158-s001.zip › ai4pain_samples/cropped/3/H1/keyframe_12.jpg]

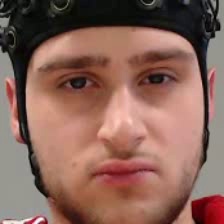

Supplement: Supplemental Information 1 [file peerj-cs-11-3158-s001.zip › ai4pain_samples/cropped/3/H1/keyframe_120.jpg]

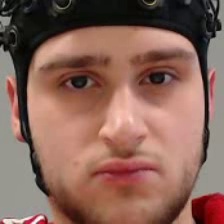

Supplement: Supplemental Information 1 [file peerj-cs-11-3158-s001.zip › ai4pain_samples/cropped/3/H1/keyframe_147.jpg]

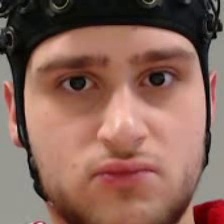

Supplement: Supplemental Information 1 [file peerj-cs-11-3158-s001.zip › ai4pain_samples/cropped/3/H1/keyframe_15.jpg]

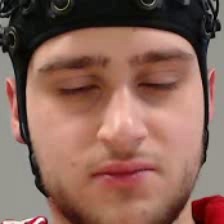

Supplement: Supplemental Information 1 [file peerj-cs-11-3158-s001.zip › ai4pain_samples/cropped/3/H1/keyframe_192.jpg]

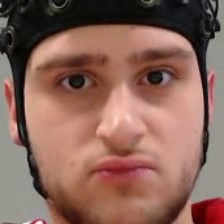

Supplement: Supplemental Information 1 [file peerj-cs-11-3158-s001.zip › ai4pain_samples/cropped/3/H1/keyframe_20.jpg]

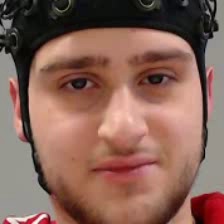

Supplement: Supplemental Information 1 [file peerj-cs-11-3158-s001.zip › ai4pain_samples/cropped/3/H1/keyframe_264.jpg]

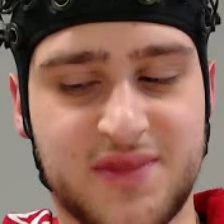

Supplement: Supplemental Information 1 [file peerj-cs-11-3158-s001.zip › ai4pain_samples/cropped/3/H1/keyframe_281.jpg]

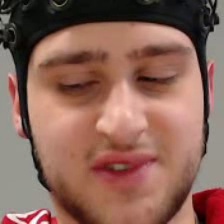

Supplement: Supplemental Information 1 [file peerj-cs-11-3158-s001.zip › ai4pain_samples/cropped/3/H1/keyframe_282.jpg]

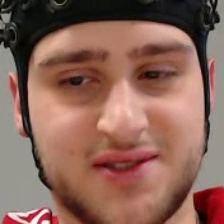

Supplement: Supplemental Information 1 [file peerj-cs-11-3158-s001.zip › ai4pain_samples/cropped/3/H1/keyframe_295.jpg]

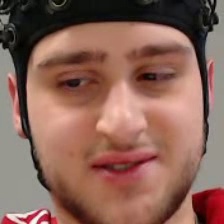

Supplement: Supplemental Information 1 [file peerj-cs-11-3158-s001.zip › ai4pain_samples/cropped/3/H1/keyframe_296.jpg]

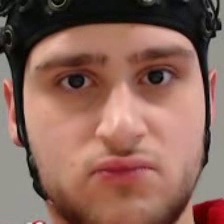

Supplement: Supplemental Information 1 [file peerj-cs-11-3158-s001.zip › ai4pain_samples/cropped/3/H1/keyframe_31.jpg]

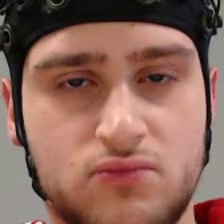

Supplement: Supplemental Information 1 [file peerj-cs-11-3158-s001.zip › ai4pain_samples/cropped/3/H1/keyframe_38.jpg]

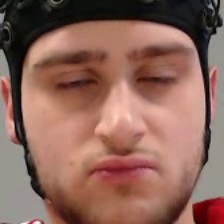

Supplement: Supplemental Information 1 [file peerj-cs-11-3158-s001.zip › ai4pain_samples/cropped/3/H1/keyframe_42.jpg]

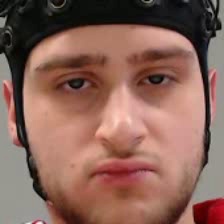

Supplement: Supplemental Information 1 [file peerj-cs-11-3158-s001.zip › ai4pain_samples/cropped/3/H1/keyframe_48.jpg]

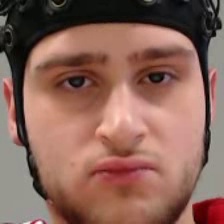

Supplement: Supplemental Information 1 [file peerj-cs-11-3158-s001.zip › ai4pain_samples/cropped/3/H1/keyframe_52.jpg]

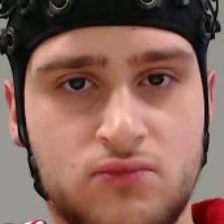

Supplement: Supplemental Information 1 [file peerj-cs-11-3158-s001.zip › ai4pain_samples/cropped/3/H1/keyframe_54.jpg]

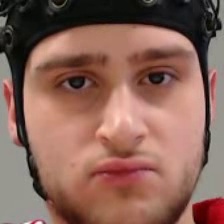

Supplement: Supplemental Information 1 [file peerj-cs-11-3158-s001.zip › ai4pain_samples/cropped/3/H1/keyframe_55.jpg]

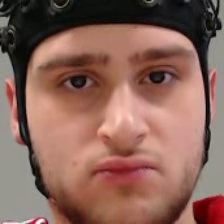

Supplement: Supplemental Information 1 [file peerj-cs-11-3158-s001.zip › ai4pain_samples/cropped/3/H1/keyframe_56.jpg]

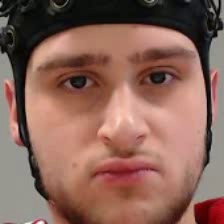

Supplement: Supplemental Information 1 [file peerj-cs-11-3158-s001.zip › ai4pain_samples/cropped/3/H1/keyframe_60.jpg]

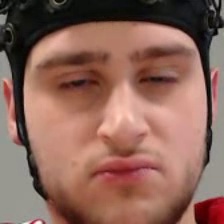

Supplement: Supplemental Information 1 [file peerj-cs-11-3158-s001.zip › ai4pain_samples/cropped/3/H1/keyframe_66.jpg]

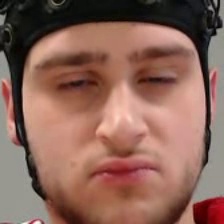

Supplement: Supplemental Information 1 [file peerj-cs-11-3158-s001.zip › ai4pain_samples/cropped/3/H1/keyframe_67.jpg]

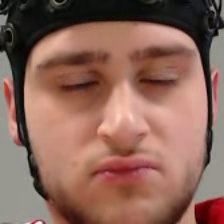

Supplement: Supplemental Information 1 [file peerj-cs-11-3158-s001.zip › ai4pain_samples/cropped/3/H1/keyframe_71.jpg]

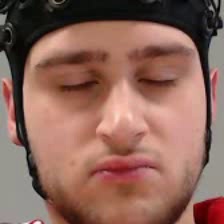

Supplement: Supplemental Information 1 [file peerj-cs-11-3158-s001.zip › ai4pain_samples/cropped/3/H1/keyframe_72.jpg]

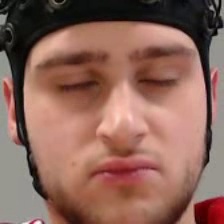

Supplement: Supplemental Information 1 [file peerj-cs-11-3158-s001.zip › ai4pain_samples/cropped/3/H1/keyframe_78.jpg]

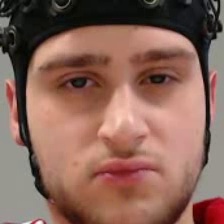

Supplement: Supplemental Information 1 [file peerj-cs-11-3158-s001.zip › ai4pain_samples/cropped/3/H1/keyframe_85.jpg]

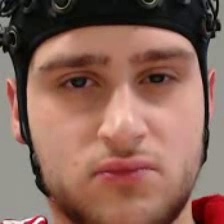

Supplement: Supplemental Information 1 [file peerj-cs-11-3158-s001.zip › ai4pain_samples/cropped/3/H1/keyframe_87.jpg]

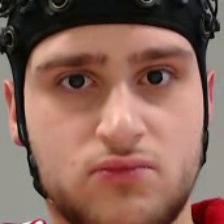

Supplement: Supplemental Information 1 [file peerj-cs-11-3158-s001.zip › ai4pain_samples/cropped/3/H1/keyframe_9.jpg]

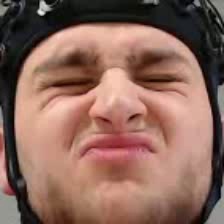

Supplement: Supplemental Information 1 [file peerj-cs-11-3158-s001.zip › ai4pain_samples/cropped/3/H2/keyframe_0.jpg]
